# Ammonium removal by erythrocyte-bioreactors based on glutamate dehydrogenase from *Proteus* sp. jointly with porcine heart alanine aminotransferase

**DOI:** 10.1038/s41598-022-09435-y

**Published:** 2022-03-31

**Authors:** Daria V. Borsakova, Larisa D. Koleva, Evgeniy S. Protasov, Fazoil I. Ataullakhanov, Elena I. Sinauridze

**Affiliations:** 1 Laboratory of Physiology and Biophysics of the Cell, Center for Theoretical Problems of Physicochemical Pharmacology, Russian Academy of Sciences, Srednyaya Kalitnikovskaya str., 30, Moscow, 109029 Russia; 2 Present Address: Laboratory of Biophysics, Dmitriy Rogachev National Medical Research Center of Pediatric Hematology, Oncology and Immunology, Ministry of Healthcare, Samory Mashela str., 1, GSP-7, Moscow, 117198 Russia; 3 Department of Physics, Lomonosov Moscow State University, Leninskie Gory, 1, build. 2, GSP-1, Moscow, 119991 Russia; 4 Department of Molecular and Translational Medicine, Moscow Institute of Physics and Technology, Institutskiy Per., 9, Dolgoprudny, Moscow Region, 141701 Russia; 5 Dmitriy Rogachev National Medical Research Center of Pediatric Hematology, Oncology and Immunology, Samory Mashela str., 1, GSP-7, Moscow, 117997 Russia

**Keywords:** Molecular engineering, Drug delivery, Drug development

## Abstract

Excessive ammonium blood concentration causes many serious neurological complications. The medications currently used are not very effective. To remove ammonium from the blood, erythrocyte-bioreactors containing enzymes that processing ammonium have been proposed. The most promising bioreactor contained co-encapsulated glutamate dehydrogenase (GDH) and alanine aminotransferase (ALT). However, a low encapsulation of a commonly used bovine liver GDH (due to high aggregation), makes clinical use of such bioreactors impossible. In this study, new bioreactors containing ALT and non-aggregating GDH at higher loading were first produced using the flow dialysis method and the new bacterial GDH enzyme from *Proteus* sp. The efficacy of these erythrocyte-bioreactors and their properties (hemolysis, osmotic fragility, intracellular and extracellular activity of included enzymes, erythrocyte indices, and filterability) were studied and compared with native cells during 1-week storage. The ammonium removal rate in vitro by such erythrocyte-bioreactors increased linearly with an increase in encapsulated GDH activity. Alanine in vitro increased in accordance with ammonium consumption, which indicated the joint functioning of both included enzymes. Thus, novel bioreactors for ammonium removal containing GDH from *Proteus* sp. are promising for clinical use, since they have a more efficient GDH encapsulation and their properties are not inferior to previously obtained erythrocyte-bioreactors.

## Introduction

Hyperammonemia (HA) is a condition characterised by an increased blood ammonium concentration and accompanying acquired liver diseases (cancer, cirrhosis, and viral diseases) and congenital enzyme deficiencies associated with ammonia metabolism^1^. Ammonium is toxic to the central nervous system, so it must be quickly removed, but the existing medications for treating HA are not very effective^2–4^. As an alternative, erythrocytes (red blood cells, RBCs) loaded with enzymes that utilize ammonium (ammocytes) were proposed for removing ammonium from the blood^5–10^. The first proposed variants of ammocytes contained either glutamate dehydrogenase^5–7^ or glutamine synthetase^8,9^ as ammonium processing enzymes. However, the time during which these ammocytes could work effectively in the body was very limited. The reason was a very low or absent rate of transport of substrates required for the reaction (α-ketoglutarate and glutamate) across the erythrocyte membrane. Analysis of the work of various types of ammocytes using specially developed mathematical models showed that the most promising are ammocytes which include a tandem of two enzymes: glutamate dehydrogenase (GDH) (reaction 1) and alanine aminotransferase (ALT) (reaction 2)^10^:

1 $${\text{AKG}} + {\text{ NH}}_{{4}}^{ + } + {\text{ H}}^{ + } + {\text{ NADPH }} \leftrightarrow {\text{GLU}} + {\text{ NADP }} + {\text{ H}}_{{2}} {\text{O,}}$$

2 $${\text{GLU}} + {\text{ PYR }} \leftrightarrow {\text{AKG}} + {\text{ ALA,}}$$

where NH_4_^+^ is an ammonium ion, AKG is α-ketoglutarate, GLU is glutamate, PYR is pyruvate, ALA is alanine, and NADP and NADPH are the oxidised and reduced forms of nicotinamide adenine dinucleotide phosphate, respectively.

In this case, the limitations associated with the transport of AKG and GLU, which exist in other ammocytes (loaded with only GDH or only glutamine synthetase), were eliminated since these metabolites were consumed and re-synthesised inside the RBC cyclically^10^. It was shown that bioreactors based on GDH and ALT, obtained by the hypoosmotic dialysis method (in dialysis bags), worked quite effectively in vitro and in vivo. However, the limiting factor for their use in the clinic was the low level of incorporation into erythrocytes of the commonly used bovine liver GDH (about 2.0–3.8% for human and mouse RBCs, respectively)^5–7,10^. In human erythrocytes, this level did not exceed 2% even with such a high activity of bovine liver GDH in the initial suspension during the enzyme encapsulation as 750 IU/ml of suspension (approximately 15 mg protein/ml of suspension)^5^. Earlier, we assumed that this could be connected with the loading method and the properties of the enzyme itself, primarily by its low specific activity, high molecular weight, and ability to aggregate with an increase in its concentration in the solution^11^. Such aggregation has been described for some animal GDHs, including bovine liver GDH, but has not been detected for any bacterial GDHs studied^12^. Therefore, we suggested using the bacterial GDH *Proteus* sp. instead of bovine liver GDH, which was previously used to obtain ammocytes. It is a NADPH-dependent enzyme with a high specific activity per 1 mg (according to the manufacturer's data, this activity is 10 times higher than for bovine liver GDH), and it could be assumed that, unlike bovine GDH, this enzyme is unable to aggregate at an increase in its concentration in solution^11–13^. As a method for enzyme loading, hypoosmotic flow dialysis was proposed, which proved to be the most effective compared to other classical laboratory methods^11^. At an osmolality of 100 mOsm/kg, this method increased the level of encapsulation of bovine GDH by two times compared to the method of hypoosmotic dialysis in bags^5–7,10^. The activity of GDH from *Proteus* sp. loaded into erythrocytes was directly proportional to the concentration of GDH in the initial suspension over a wide concentration range, confirming that this enzyme did not aggregate with increasing concentration. This allowed an approximately 20-fold increase in its specific enzymatic activity in ammocytes (at the GDH concentration in the initial suspension of 1.4 mg/ml)^11^. However, in this earlier study, encapsulation into RBCs of GDH (from bovine liver or *Proteus* sp.) using different methods was studied only in the absence of ALT.

The novelty of this work lies in the fact that new ammoocytes containing ALT and GDH with an increased degree of encapsulation in erythrocytes were prepared for the processing of ammonium. To do this, a more efficient method of enzyme incorporation (the method of flow dialysis), as well as a new enzyme (bacterial GDH from *Proteus* sp.), which is not aggregating in solution when its concentration increases, were used. We showed that a presence of ALT does not prevent the encapsulation of *Proteus* sp. GDH into erythrocytes. Besides, the properties of new bioreactors were studied for the first time. This study included an ammocyte filterability parameter that had never been studied before for any erythrocyte-bioreactors. All the studied properties were not inferior to the properties of various erythrocyte-bioreactors previously obtained in other works^5–8,11^. For the new bioreactors, ammonium removal rates from the medium in vitro were also measured, which were proportional to the GDH activity in RBCs. The amount of ammonium consumed was always equal to the amount of formed alanine, which confirms the coordinated work of both enzymes included. Our results confirmed the possibility of using GDH from *Proteus* sp. to increase the efficiency of GDH encapsulation into RBCs without loss of cell quality. This makes the new ammoocytes, unlike all other ammocytes, promising for clinical use.

## Results and discussion

### Efficiency of enzymes’ encapsulation and cell recovery

A detailed description of the conditions for the encapsulation of enzymes in RBCs using flow dialysis is given in the “Materials and methods” section. The efficiency of the encapsulation procedure was estimated using two parameters: the percentage of encapsulated enzymes (*E*, %) and cell recovery (*C*, %) (Eqs. 5 and 6 in “Materials and methods”, respectively). A comparison of the results of this and previous studies is presented in Table 1.

**Table 1:** Efficiency of encapsulation of ALT and different types of GDH, as well as cell recovery for different methods of enzyme loading.

Encapsulated enzyme	GDH from bovine liver		GDH from *Proteus* sp.		ALT from porcine heart	
Method of encapsulation	Dialysis in bag	Flow dialysis	Dialysis in bag	Flow dialysis	Dialysis in bag	Flow dialysis
Encapsulation (%)	1.8 ± 0.8 (n = 8)^5^	–	–	–	–	–
	2.28 ± 0.93 (n = 7)^11^	4.70 ± 1.41 (n = 9)^11^	–	12.20 ± 4.25 (n = 5)^11^	–	–
	2.20 ± 0.82 (n = 10)^10^		–		11.69 ± 6.39 (n = 10)^10^	–
				13.5 ± 3.6 (n = 17) (this study)		28.40 ± 5.40 (n = 17) (this study)
Cell recovery (%)	65.1 ± 4.5 (n = 8)^5^		–	–	–	–
	44.0 ± 4.8 (n = 7)^11^	66.1 ± 12.0 (n = 9)^11^		63.7 ± 2.2 (n = 5)^11^	–	–
	43.3 ± 12.0 (n = 10)^10^			–	43.3 ± 12.0 (n = 10)^10^	–
				69.0 ± 8.6 (n = 19) (this study)		69.0 ± 8.6 (n = 19) (this study)

Mean values and standard deviations (mean ± SD) are presented for human erythrocytes.

In studies^5,11^ GDHs from various species were incorporated into erythrocytes without ALT. In the present study and work^10^, various GDHs were incorporated into erythrocytes along with ALT.

There is a significant difference in the encapsulation of GDH (any type) and ALT obtained in these studies (ANOVA, p < 0.05). This difference can be caused by differences in the molecular weight and size of enzyme molecules, which affect the probability of their penetration through the pores that arise in the RBC membrane under hypoosmotic conditions (average pore size 8–10 nm)^14^. The ellipsoidal bovine liver GDH molecule has a molecular weight of 340 kDa, a length of 13.3 nm and a diameter of 4.3 nm^13,15^, while the globular ALT molecule has a molecular weight of only 150 kDa and an approximate diameter of 4 nm^16^. Molecules GDH *Proteus* sp. and bovine liver GDH are very similar in shape. The GDH molecule from *Proteus* sp. also has an ellipsoidal shape and a molecular weight of about 300 kDa^17^. As a result, the ALT molecule has the higher probability of penetrating the erythrocyte membrane. The results of this study are consistent with previous results obtained by the same flow dialysis method^11^. The encapsulation of *Proteus* sp. GDH using flow dialysis (13.5%) is almost 3 times higher than that for bovine liver GDH using the same method, due to its inability to aggregate^11^, and about 6 times higher than encapsulation of bovine GDH found in previous works, where dialysis in bags was used for encapsulation^5,10,11^. The presence of the second enzyme (ALT) does not decrease the efficiency of GDH encapsulation by flow dialysis (compare this study and study^11^ in Table 1). Cell recovery was also enhanced using flow dialysis, consistent with previous results obtained using standard medical hemodialyzers^18^.

### Changes in the properties of erythrocytes after flow dialysis

Since it is assumed that ammocytes are subject to further clinical use, it is necessary to determine their approximate shelf life after receiving using flow dialysis. The following properties of ammocytes in comparison with native RBCs were studied at different storage times of 10% cell suspensions at + 4 °C: erythrocyte indices, hemolysis, osmotic fragility, as well as intracellular and extracellular activity of GDH and ALT. All these parameters were measured within 6 days of storage. In addition, deformability (as filterability) was measured in freshly prepared RBCs and ammocytes, as well as in ammocytes after 2 h of incubation at room temperature.

### Erythrocyte indices

Standard erythrocyte indices [mean erythrocyte volume, mean content and mean concentration of hemoglobin (Hb) in it] always change after cells' exposure to hypoosmotic stress. After opening the pores in the erythrocyte membrane, part of the intracellular contents, including low molecular weight metabolites of glycolysis, as well as part of hemoglobin and glycolytic enzymes, leave the erythrocyte into the external environment. As a result, the volume of the cell and the total content of Hb in it decrease somewhat. There is also a decrease in the mean concentration of hemoglobin in the cell, but it is less pronounced, since along with a decrease in the Hb content, the cell volume also decreases^5,7,11^.

As mentioned above, similar changes are observed with any method of incorporating enzymes into erythrocytes, which uses a hypoosmotic effect on cells. However, it is also well known, that with such a degree of change in erythrocyte indices, there is no deterioration in the state of erythrocytes, which would lead to a reduction in their life in the bloodstream^19^. The main functions of the erythrocytes are to carry oxygen to organs and tissues and maintain their own vitality, which allows them to successfully perform this function. A decrease in the content of Hb and glycolytic enzymes in the erythrocyte by 30–50% cannot significantly affect the overall oxygen transport function of erythrocytes. Since the proportion of ammocytes transfused to the patient will not exceed 10–20% of all RBCs, then even a 50% decrease in Hb in them will lead to a general decrease in Hb in blood by no more than 10%. Additional RBCs transfusion may be required for a patient if the Hb level in his/her blood decreases by much more than 10% (from about 130 g/l to a level below 70–60 g/l)^20^.

A decrease in the Hb content in an erythrocyte does not affect its survival in the bloodstream, but a decrease in glycolityc enzymes could affect the survival of an erythrocyte, since glycolysis is the only system that provides the erythrocyte with energy. However, even a 50% decrease in the concentration of some glycolytic enzymes during the hypoosmotic dialysis procedure will not lead to a decrease in the efficiency of glycolysis in the erythrocyte, because it was shown that all glycolytic enzymes are contained in it in a large excess^21^.

**Figure 1:**
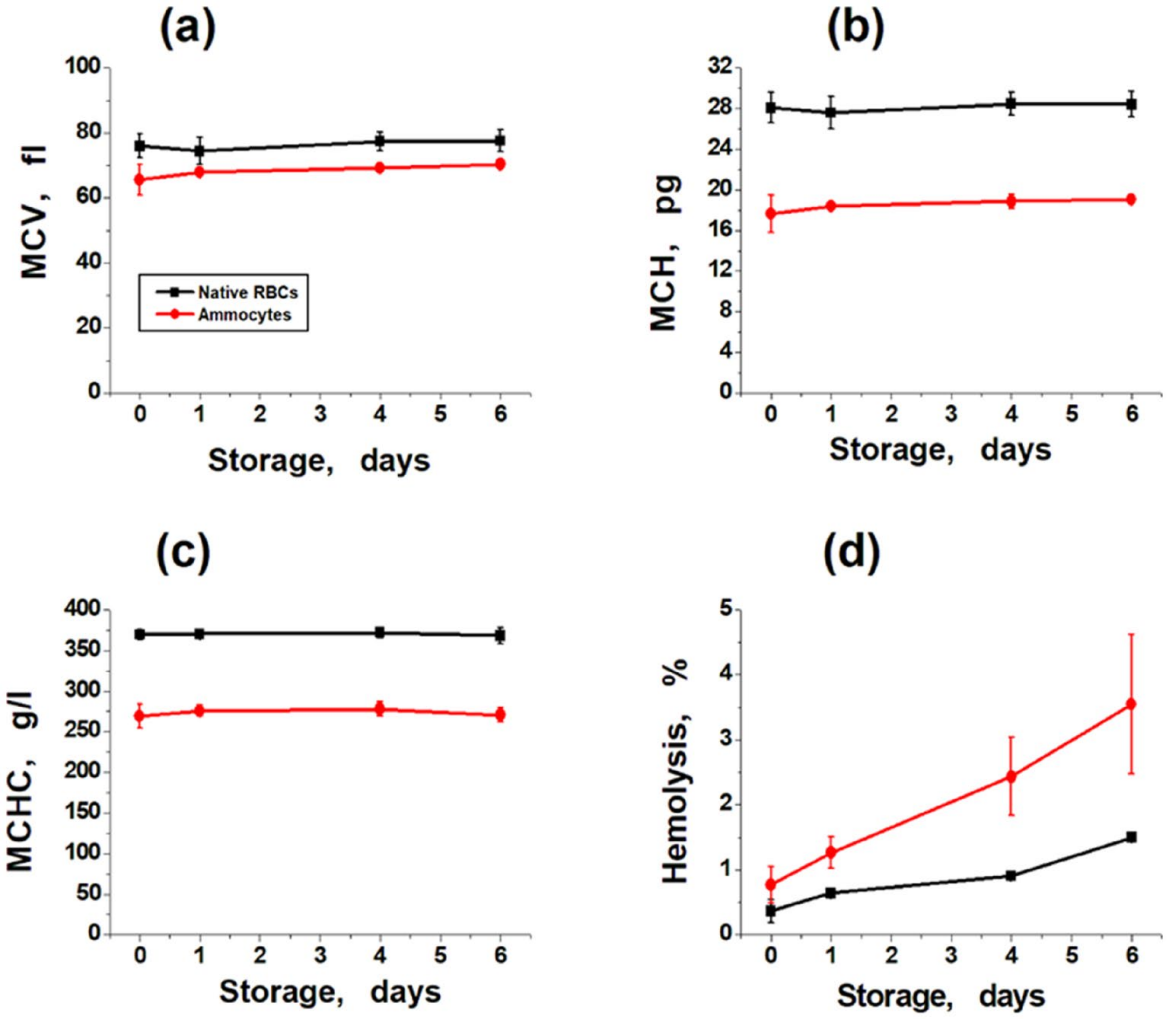
Erythrocyte indices and hemolysis of native RBCs and ammocytes during storage. Suspensions of native RBCs (black symbols) and ammocytes (red symbols) with a hematocrit of 10% were stored at + 4 °C for 6 days. (**a**) Mean cell volume (fl). (**b**) Mean cell hemoglobin content (pg). (**c**) Mean cell hemoglobin concentration (g/l). (**d**) Hemolysis of ammocytes and native cells during storage. Mean values ± SD are presented. For all erythrocyte indices, n = 5 (except for day 0, where n = 7). For hemolysis of ammocytes n = 4, except for day 0 (n = 5), for hemolysis of native cells n = 12, except for day 0 (n = 7).

Erythrocyte indices of ammocytes containing GDH from *Proteus* sp + ALT, obtained by flow dialysis, are shown in Fig. 1a–c. They changed slightly during flow dialysis procedure, but then remained constant during storage. The degree of their change did not exceed the change in similar indices in other previously published studies^5,7,11^, so these parameters were not limiting for 6 days of storage.

### Hemolysis of native erythrocytes and ammocytes

The hemolysis rate of ammocytes (Fig. 1d) was higher than that of the initial erythrocytes, suggesting that the osmotic resistance of ammocytes is reduced compared to native erythrocytes. Thus, their shelf life had to be limited to 1–2 days (depending on the dose of ammocytes to be transfused). At longer storage periods, the ammocytes must be additionally washed before transfusion.

### Osmotic fragility

**Figure 2:**
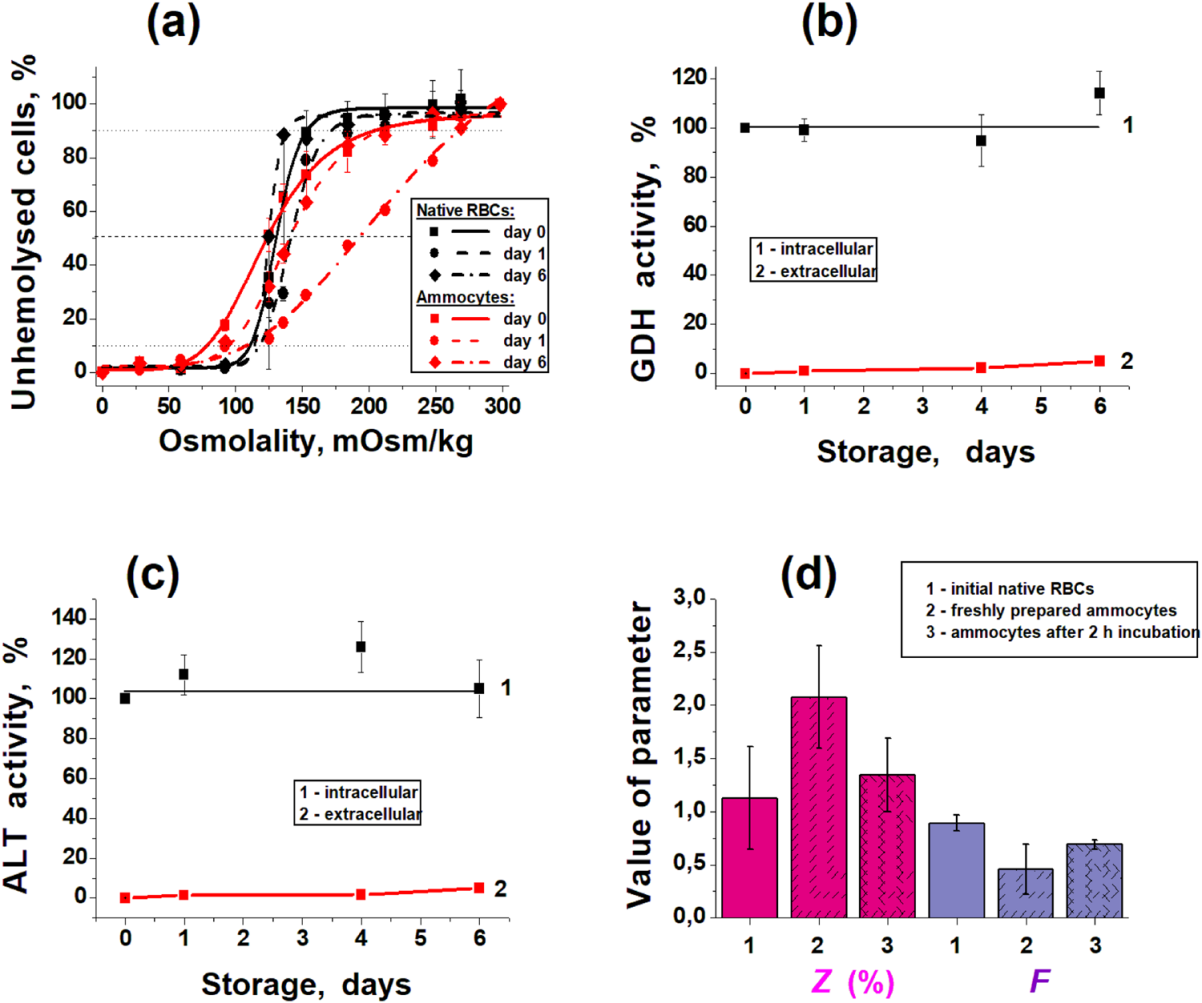
Properties of ammocytes and native RBCs. (**a**) The curves of osmotic fragility of ammocytes (red lines) and native RBCs (black lines) during storage at + 4 °C in 10% suspensions, expressed as the percentage of unhemolysed cells in dependence on solution osmolality. The measurements were carried out at day 0 (solid lines), day 1 (dash lines), and day 6 (dash-dot lines). Means ± SD are presented, n = 4. (**b**,**c**) GDH and ALT activities, respectively, inside the cells (1) and in the extracellular medium (2) as the percent of intracellular activity at day 0. Means ± SEM are presented, n = 8. (**d**) Filterability of native RBCs (1), freshly prepared ammocytes (2), and ammocytes, incubated for 2 h at room temperature (3), expressed as the fraction of cells unable to pass through the filter *Z* (%), or as index of filterability, *F*. Means ± SD are presented, n = 7.

Whether cells (native RBCs and ammocytes) will hemolyse in the bloodstream after transfusion, can be indirectly determined by their osmotic fragility. This parameter characterizes the resistance of cells to a decrease in the osmolality of the medium (to osmotic stress). It was changed in freshly prepared ammocytes compared to native RBCs (Fig. 2a), which was consistent with results previously obtained for carrier erythrocytes in other studies^5,7,8,11^.

From the averaged curves of osmotic fragility shown in Fig. 2a, it can be seen that the osmotic fragility of ammocytes characterized by H_50_, osmolality of the medium, at which 50% of cells are lysed (dashed line), during the entire storage period exceeded the osmotic fragility of native erythrocytes during the same storage period. The difference between groups for this parameter was 6 mOsm/kg on day 0, 54 mOsm/kg on day 1, and decreased again to 16 mOsm/kg on day 6 of storage. This is consistent with a higher rate of ammocyte hemolysis during storage. The width of the distribution of erythrocytes by osmotic fragility (W, the difference in the osmolality of the medium in which 10% and 90% of the cells were lysed, dotted lines) was also significantly higher in ammocytes than in native erythrocytes on any day of storage. However, in contrast to H_50_, this difference gradually decreased during storage, amounting to 110, 106, and 93 mOsm/kg for storage days 0, 1, and 6, respectively. Such changes in these parameters during the storage of native erythrocytes and ammocytes can be explained by the fact that the suspension of ammocytes contains a certain proportion of cells spoiled during the procedure, which undergo more rapid destruction with a decrease in the osmolality of the medium than native erythrocytes. However, during storage, these cells are destroyed quite quickly, and the remaining intact ammocytes approach the properties of the original native erythrocytes.

### Changes in the concentration of enzymes inside ammocytes during storage

The leakage of enzymes (GDH and ALT) from ammocytes for 6 days of the storage did not exceed 10% (apparently due to hemolysis of some cells) and did not affect the intracellular enzymes activities (Fig. 2b,c). This confirms that both enzymes inside erythrocytes are sufficiently stable and protected from damaging external influences.

### Filterability

The ability of erythrocytes to change shape to pass through small capillaries in the spleen directly determines their half-life in the bloodstream. This property also determines the ability of cells to pass through the small pores of the artificial filter. The deformation capacity of cells in the test of filterability is characterised by two parameters: the filterability index (F), determined by Eq. (9), and the proportion of cells in the suspension that cannot pass through the filter pores (Z), determined by Eqs. (10) and (11) (see section “Materials and methods”). The deformability of erythrocyte-bioreactors has never been studied before. This was done for the first time in this work.

Whereas the filterability index F of fresh native cells is near 1 (which means that cells pass the filter pores almost the same time as the buffer), freshly prepared ammocytes have twice decreased filterability index F and twice increased value of Z compared to native RBCs (Fig. 2d). However, these parameters of ammocytes tend to improve after 2 h incubation at room temperature in a solution supplied with glucose, adenine and albumin (Fig. 2d). This suggests that the metabolism of ammocytes is actively working and gives hope that when transfused into the body, ammocytes will fully restore their ability to deform.

### Efficiency of ammonium removal by ammocytes in vitro

**Figure 3:**
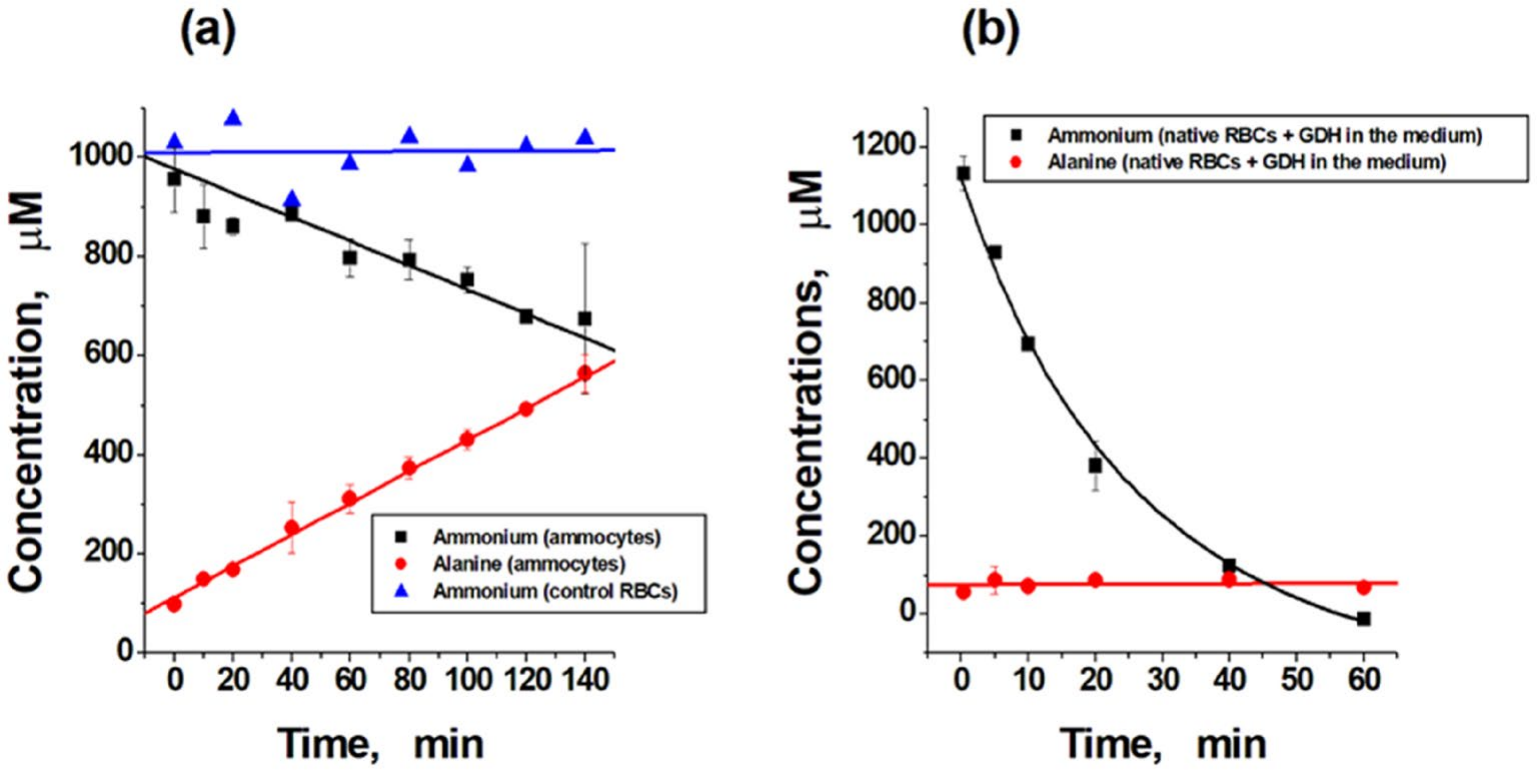
Typical kinetics of ammonium consumption and alanine production in the presence of ammocytes or free enzymes in vitro. Measurements were carried out in the presence of ammocytes from one of the donors (suspension hematocrit 23%, temperature 25 °C, and 1 mM of added NH_4_Cl). (**a**) Kinetics of ammonium consumption (black line) and alanine formation (red line) in the presence of ammocytes (activities of GDH and ALT in the suspension are equal to 0.57 IU/ml and 6.7 IU/ml, respectively), as well as the concentration of ammonium in the control in the presence of native cells (blue line). (**b**) Kinetics of ammonium and alanine concentrations in the presence of native RBCs and added to the external medium GDH (0.2 IU/ml of solution). Mean values ± SD are presented, n = 2.

The efficiency of ammonium removal by the obtained erythrocyte-bioreactors was studied in vitro. A suspension of these bioreactors with added NH_4_Cl (1 mM) was incubated for 120–140 min at 25 °C. Samples were collected every 20 min and ammonium and alanine were measured. Typical kinetics curves for one of the donors are shown in Fig. 3a. As a control, the ammonium removal in the presence of native RBCs in medium with added NH_4_Cl was measured. There was a linear decrease in ammonium concentration and comparable alanine production in the presence of ammocytes, along with no change in ammonium concentration in the presence of native erythrocytes. This kinetics differed significantly from the kinetics of removing ammonium from the medium where native RBCs and free GDH (without ALT) were present (Fig. 3b).

All concentrations in Fig. 3 are given in mmol per 1 l of sample suspension, i.e., they include the concentration of each metabolite in cells and supernatant (in appropriate proportions). At the same time, the concentration of ammonium in the suspension is the same everywhere, since ammonium very quickly penetrates into the erythrocyte and its concentration in the cell and the supernatant is equalized, and the concentration of alanine in the cells and in the external environment can be different. The ratio of alanine concentrations in ammocytes and in the external environment is determined by the rate of alanine production and the rate of its transport across the erythrocyte membrane. In this work, only the total concentration of alanine in the suspension was measured, however, the question of how much its concentration inside erythrocytes can increase during the operation of bioreactors is very important. In our previous work^10^, the accumulation of alanine in the erythrocyte was calculated using a mathematical model in which the transport of alanine was presented in a very simplified manner as a linear process proportional to the gradient of the concentration of alanine inside and outside the erythrocyte. Calculations using such a simplified model showed that alanine is not able to accumulate inside the cell in high concentrations. During the operation of the bioreactor, its concentration inside the cell quickly reaches a stationary level, which does not exceed 0.84 mmol/l_RBCs_ (at activity of GDH and ALT in RBCs equal to 8 and 40 IU/ml_RBCs_, respectively). At the moment, we are using a refined mathematical model of ammocytes, in which the transport equations for all metabolites are written in accordance with their real mechanisms^22^.

**Figure 4:**
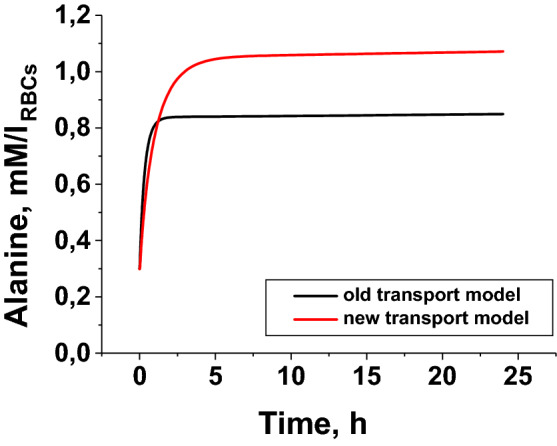
Accumulation of alanine in ammocytes. Theoretical simulation of alanine accumulation inside RBCs during the work of ammocytes, calculated using simplified equations for the alanine and other metabolites transport (black line)^10^ and a refined model using equations describing the real transport mechanisms (red line)^22^. Activity of GDH from *Proteus* sp. and ALT was 8 and 40 IU/ml_RBCs_, respectively. The ammonium concentration was kept constant at 0.5 mM.

It is well known that alanine can penetrate the erythrocyte membrane using two transport systems: the ASC system—saturable, dependent on Na^+^, which is described by the Michaelis–Menten equation, and the L system, which transfers alanine linearly, in proportion to its concentration, and does not depend on Na^+^. Despite the fact that the influx (or efflux) of alanine here increases linearly, in proportion to its concentration, this is not a process of simple diffusion, but the work of a certain carrier of alanine, therefore, this process can be called "diffusion" only conditionally. In several works devoted to the experimental study of the alanine transport, the kinetic constants of both these systems were measured^23–26^. The equation for the rate of alanine transport can be written as follows:

3 $${\text{V}}_{{{\text{transp}}.}} = \left[ {({\text{V}}_{{{\text{transp}}.{\text{ max}}}} \times \, } \right[{\text{ALA}}\left] {)/({\text{K}}_{{\text{M}}} + \, } \right[{\text{ALA}}\left] ) \right] \, + {\text{k}}_{{\text{d}}} \times \left[ {{\text{ALA}}} \right],$$

where V_transp. max_ is the maximum rate of the alanine influx into RBCs, or efflux into external medium, if the concentration of [ALA] in or out RBC is used, respectively. K_M_ is the Michaelis constant for the carrier of the ASC system, and k_d_ is the permeability coefficient of the carrier of the L system. The total rate and direction of alanine transport will be determined by the difference in the rates of its entry and exit from the erythrocyte. Calculation of the accumulation of alanine in the bioreactor according to the refined mathematical model^22^ somewhat changed the value of the steady-state concentration, which is achieved in the erythrocyte during the operation of the bioreactor, but did not fundamentally change the previously made conclusion that alanine is not able to accumulate in the cell in concentrations that could threaten its integrity due to an increase in osmotic pressure (Fig. 4). The obtained maximum level of the steady-state alanine concentration after 24 h was about 1.15 mM, which is clearly insufficient for destruction of the erythrocyte due to an increase in intracellular osmolality (which is possible only when the alanine concentration into RBCs is more than 50–60 mM)^10^.

**Figure 5:**
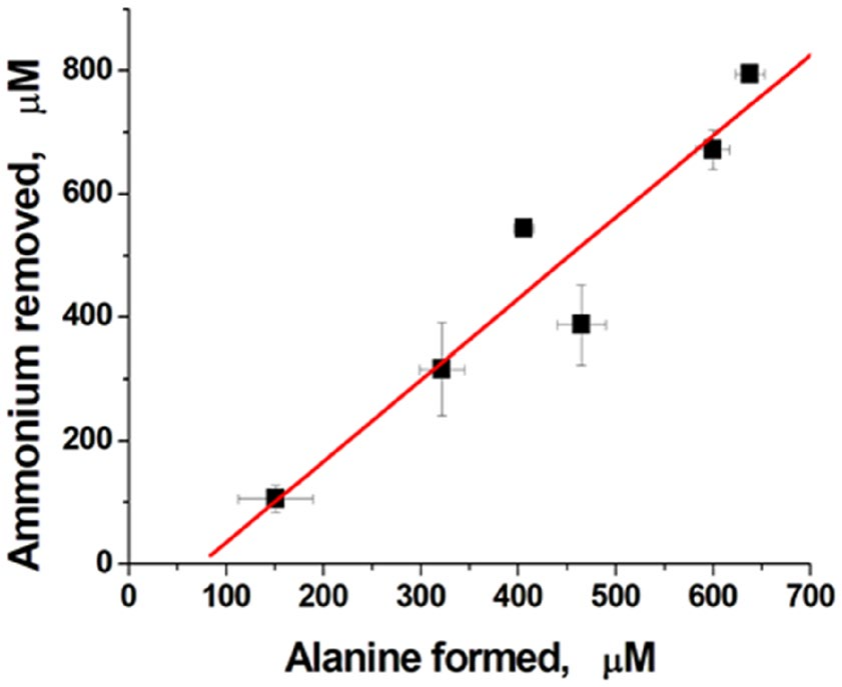
The relationship between ammonium consumption and alanine formation. The measurements were carried out for different donors with different activity of encapsulated enzymes after incubation of a suspension of ammocytes (hematocrit 23%) in a sealed cuvette (3 ml) in vitro at 25 °C for 120–140 min in the presence of 1 mM added NH_4_Cl. Means ± SD are presented. For all donors, n = 2, except for the first point, where n = 4.

**Figure 6:**
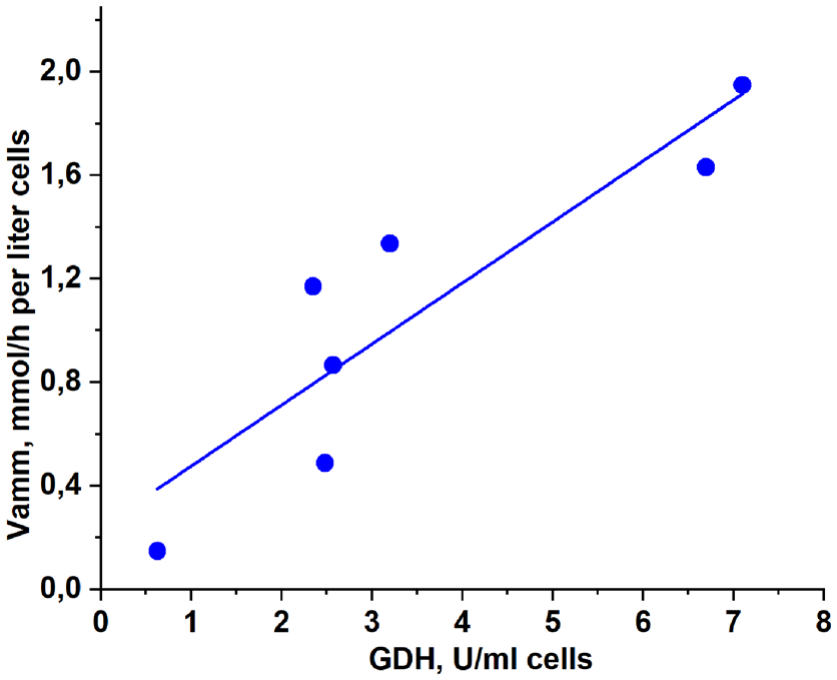
Dependence of the ammonium consumption rate in the suspension of ammocytes on the GDH activity. Suspension hematocrit 23%, temperature 25 °C, 1 mM NH_4_Cl and 10 mM PYR added. The ALT activity in all experiments was 2–3 times higher than the corresponding GDH activity.

The averaged results showed that the removal of ammonium by the bioreactors was accompanied by the formation of a proportional amount of alanine (Fig. 5). This phenomenon suggests that GDH and ALT function in tandem and ammonium is converted to alanine according to Eqs. (1) and (2). It was also shown that the rate of ammonium removal from the medium in vitro in the presence of ammocytes was dependent on the activity of GDH in ammocytes (Fig. 6). The increase in the intracellular GDH concentration caused the increase in this rate. Consequently, the action of ammocytes is dose-dependent and can be controlled during clinical use.

Theoretically, accumulation of alanine in ammocytes can inhibit ALT reaction and affect the efficiency of ammonium consumption by the erythrocyte-bioreactors. However, our results show that even in in vitro experiments with permanent significant alanine accumulation it does not affect the rate of ammonium consumption (Figs. 3, 5, 6). Consequently, we should not expect an influence of alanine on ammonium consumption rate under in vivo conditions, when alanine produced in the ammocytes is transported to blood plasma (Fig. 4).

Finally, we would like to note that preclinical studies of a biomedical product such as erythrocyte-bioreactors are always a complex issue. Testing such a biomedical product will undoubtedly require studies of dose, sterility, stability, and other necessary parameters of the product for transfusion. Also, enzymes, especially heterologous, that are used for preparation of erythrocyte-bioreactors, can cause adverse immune reactions in case of administration to patients. However, entrapment of enzymes into erythrocytes prevents interaction of the enzymes with recipient immune system. That protects the enzymes from destruction by recipient’s immune system and prevents adverse immune reactions of recipient to the encapsulated enzymes. No significant adverse immune reactions to different erythrocyte-bioreactors were reported in in vitro experiments and in clinical studies^27–29^. Anyway, the ammocytes certainly must be tested on safety for humans. However, this is beyond the scope of our study.

## Conclusions

The main problem of all previous work regarding the production of effective erythrocyte-bioreactors that remove ammonium from the bloodstream was the inability to achieve a significant incorporation of the enzyme that utilizes ammonium (bovine liver GDH) in erythrocyte-bioreactors^5,10,11^. This incorporation was a maximum of 2% when used the hypoosmotic dialysis method^5,10,11^ and of about 5% using flow dialysis method^11^ (Table 1).

In this work, a new type of ammocytes with increased GDH loading was obtained, which contained simultaneously bacterial GDH from *Proteus* sp. and ALT of porcine heart. To produce these bioreactors, the most effective method of enzyme incorporation (flow hypoosmotic dialysis) and the most promising ammonium processing enzyme (bacterial GDH from *Proteus* sp.) were used. Using bacterial GDH from *Proteus* sp. allowed us to achieve incorporation efficiency of about 13.5% for GDH when it was incorporated into erythrocytes in combination with ALT (Table 1).

As previously shown, the presence of ALT avoids the limitations associated with the poor permeability of the substrates of these reactions (α-ketoglutarate and glutamate) through the erythrocyte membrane^10^. On the other hand, we showed that the reaction product alanine did not accumulate in the erythrocyte in high concentrations during the bioreactor operation, which would be dangerous for the survival of cells in the bloodstream.

This work also studied the properties of new bioreactors. It was shown that they are not inferior to erythrocyte-bioreactors previously obtained in properties such as erythrocyte indices and osmotic fragility^5,7,8,11^. In addition, for the first time, the ability of the resulting bioreactors to deform (by their filterability through an artificial membrane filter with pores with a diameter of 3.5 μm) was studied. The filterability of freshly prepared erythrocyte-bioreactors decreased by about 2 times compared to the original native erythrocytes, but was restored to about 70–80% of the normal value after 2-h incubation at room temperature in an isotonic medium containing glucose, albumin, MgCl_2_ and adenine.

The obtained ammocytes were able to remove ammonium from the medium in vitro, and their efficiency was directly proportional to the activity of GDH in cells. The enzymes were confirmed to work in tandem because the amount of alanine produced during the reactions was strictly proportional to the amount of ammonium consumed. The fact that the used bacterial GDH does not aggregate with an increase in its concentration in solution allows us to hope that an increase in the encapsulation of this enzyme can provide the necessary therapeutic efficiency of these bioreactors for their clinical use.

A separate study of changes in the properties of the obtained ammocytes during 6 days of storage at + 4 °C showed that during this time, the activities of both enzymes inside erythrocytes do not change and are not detected in the extracellular medium. The rate of hemolysis of bioreactors during storage was higher than that of the original native erythrocytes, so, additional washing of bioreactors that were stored for more than 1 day before administration to the patient may be required.

The removal of ammonium by new bioreactors was investigated in this work only under in vitro conditions. This somewhat limits the significance of the results obtained. However, the human erythrocytes we used to prepare new ammocytes cannot be directly transfused into mice because of their large size. There is a big difference between manipulation with human and animal erythrocytes, so the results of preclinical studies in animals may be useless for further human application. Since the study of HA treatment in a mouse model is beyond the scope of this work, in our case, the only result of such in vivo experiments may be confirmation that the obtained ammocytes are able to work in the body. But we have previously shown this for a tandem of enzymes (GDH from bovine liver and ALT) encapsulated in erythrocytes in a mouse model of hyperammonemia in vivo^10^. There is no reason to believe that replacing the source of the GDH enzyme will affect the ability of ammocytes to work in vivo, while maintaining their ability to work in vitro, since the properties and regulation of GDHs from bovine liver and *Proteus* sp. are very similar^17,30^.

The refined mathematical model of new bioreactors^22^ predicts that under physiological conditions, the maximum rate of ammonium removal from the bloodstream using these bioreactors corresponds to 3.5 mmol/h × l of erythrocyte-bioreactors (at 100% hematocrit). Thus, the ammonium removal rate in a patient with a blood volume of 5 l who received 200 ml of such bioreactors will be 3.36 mmol/l of blood per day, which is almost 6 times higher than that of the most effective drugs that exist today (maximum 0.6 mmol/l of blood per day)^10^. Such bioreactors can remove 18.6 mmol of ammonium from the body per day. The question remains whether it is possible to achieve in ammocytes the specific concentration of GDH, which could provide the required maximum rate of ammonium consumption at reasonable concentrations of enzymes in the medium during encapsulation. This requires further experimental and theoretical research.

## Materials and methods

### Ethics statement

This study was approved by the Independent Ethical Committee of Dmitriy Rogachev National Medical Research Center of Pediatric Hematology, Oncology, and Immunology, Ministry of Healthcare of Russia, Moscow (Permit Number: 4e/3-19 from 28.05.2019).

The blood of healthy donors was received at the station of blood transfusion and was used without authentication. All participants gave their informed consent for experiments performance. All methods were carried out in accordance with the approved guidelines and regulations.

### Materials

Alanine aminotransferase (glutamic–pyruvic transaminase, EC 2.6.1.2) from porcine heart; rabbit muscle l-lactate dehydrogenase (EC 1.1.1.27); recombinant alanine dehydrogenase (ADH, EC 1.4.1.1); glucose; adenine; inosine; alanine; nicotinamide adenine dinucleotide (NADH) and nicotinamide adenine dinucleotide phosphate (NADPH); phosphate buffered saline (PBS) containing 10 mM sodium phosphate buffer, 138 mM NaCl and 2.7 mM KCl (pH 7.4); α-ketoglutarate; pyridoxal 5′-phosphate; sodium pyruvate, ethylenediaminetetraacetic acid (EDTA); bovine serum albumin (BSA), 4-(2-hydroxyethyl)-1-piperazineethanesulfonic acid (HEPES), and inorganic salts were purchased from Sigma-Aldrich (St. Louis, MO, USA). l-glutamate dehydrogenase from *Proteus* sp. was purchased from Toyobo Co. Ltd (Osaka, Japan).

For production of slow-volume dialyzers, the polysulfone fibers from Fresenius pediatric dialyzers were used (standard dialysis cartridges Fresenius HF 80S).

### Isolation and washing of erythrocytes

Blood from healthy donors was collected by puncture of the cubital vein into standard Vacuette vacuum tubes (Greiner Bio-One GmbH, Austria) with 3.2% (0.109 M) trisodium citrate dehydrate (citrate/blood ratio was 1/9). Erythrocytes were separated by centrifugation at 1000*g* for 8 min. Plasma and buffy-coat were removed. Erythrocytes were washed 3 times in a fourfold volume of PBS solution, followed by centrifugation for 8 min at 1000*g*.

### Hypoosmotic dialysis in small-volume dialyzer

Since the minimal volume of a medical (pediatric) dialyzer is about 20 ml, small-volume dialyzers were developed to reduce reagent consumption during enzymes’ encapsulation by hypoosmotic flow dialysis^11^.

**Figure 7:**
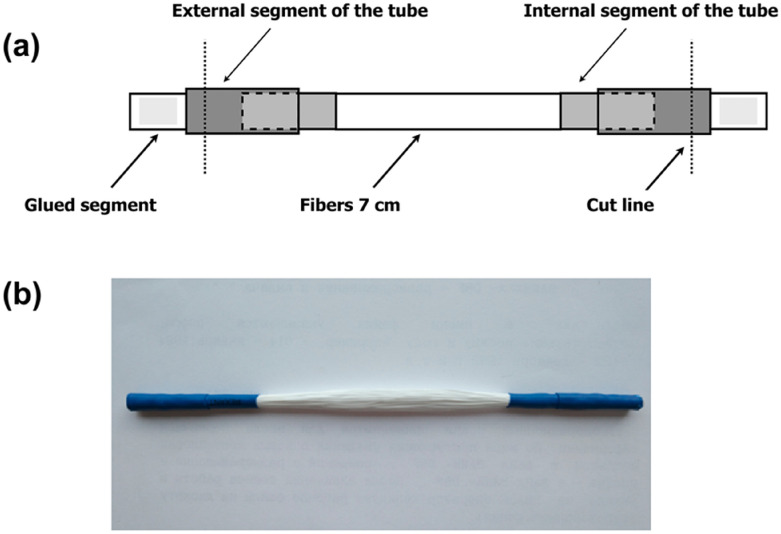
Scheme (**a**) and photo (**b**) of a small-volume dialyzer.

This small-volume dialyzer is a bundle of semi-permeable Fresenius polysulfone fibers with a final working length of 7 cm. To standardize all dialyzers, a bundle of dialysis fibers (fiber wall thickness 30–35 µm, 200 µm total fiber diameter, and 5–8 nm pore diameter) was cut from a standard dialysis cartridge Fresenius HF 80S. The fibers were weighed to obtain a bundle mass of 0.237–0.240 g, which corresponds to approximately 450–455 fibers. A detailed method of dialyzers manufacture and their leak testing is described in Ref.^11^. The volume of the dialyzer is 0.85–1 ml (Fig. 7).

To study the ammonium removal rates at different GDH and ALT activities included into RBCs, different volumes of GDH from *Proteus* sp. (NADP-dependent, 1950 IU/ml aqueous solution) and ALT from the porcine heart (200 IU/ml in 0.1 M Tris buffer, pH 7.4) were added to the suspension of washed RBCs. The final volume and hematocrit of the suspension were 2 ml and 65 ± 5%, respectively. The resulting mixture was passed at a rate of 0.2 ml/min (using a Perista pump, Atto, Japan) through a small-volume dialyzer, which was immersed in a stirred hypoosmotic solution (5 mM KH_2_PO_4_/K_2_HPO_4_, 2 mM MgCl_2_, 5 mM glucose, 37 mM NaCl, 90 mOsm/kg, pH 7.4). The volume ratio of the suspension and the hypoosmotic solution was 1:100. The dialysis time was about 15 min. Before re-sealing of RBCs after hypoosmotic dialysis, solutions of NADPH and pyridoxal 5′-phosphate were added to the dialysed cells at final concentrations of 0.3 and 0.2 mM, respectively. Sealing solution (1 M NaCl, 50 mM KH_2_PO_4_/K_2_HPO_4_, 5 mM ATP, 50 mM glucose, 50 mM sodium pyruvate, pH 7.4, 2170 mOsm/kg) was added to the dialysate so that the final suspension osmolality changed to 300–350 mOsm/kg.

The addition volume was calculated by the Eq. (4):

4 $${\text{V}}_{{{\text{hyper}}}} \left( {{\text{ml}}} \right) \, = {\text{ V}}_{{{\text{dialysate}}}} \times \left( {{35}0 \, {-}{\text{ S}}_{{{\text{dialysate}}}} } \right)/\left( {{217}0 \, {-}{ 35}0} \right),$$

where V_hyper_ is the hyperosmotic solution volume that should be added (in ml), S_dialysate_ is the dialysate osmolality, which is considered equal to the hypoosmotic solution osmolality (90 mOsm/kg), 350 is the desired final osmolality for the resulting suspension (in mOsm/kg), 2170 is the hyperosmotic solution osmolality (in mOsm/kg), V_dialysate_ is the measured volume of the dialysate obtained (in ml).

Then the suspension was incubated for 30 min at 37 °C, and the resulting ammocytes were washed 3 times with a fourfold volume of PBS solution by centrifugation at 1000*g* for 8 min.

### Measurement of GDH and ALT activity

The activity of enzymes in the initial suspension of RBCs with added enzymes and in the resulting suspension of ammocytes was measured in lysates, prepared by adding distilled water to the sample at a sample/water volume ratio of 1:19. Lysates with too high enzyme activity were diluted twice for correct measurement. Methods for measuring both enzymes were adapted to measure small sample volumes with a microplate rider. The activity of each enzyme in the lysates was measured spectrophotometrically at 30 °C using an Anthos Zenyth 340RT photometric microplate reader (Biochrom, Ltd., Cambridge, UK) at λ = 340 nm.

GDH activity was measured according to the manufacturer's protocol (Toyobo, Japan)^31^ with small modifications. The KH_2_PO_4_ buffer was used instead of Tris–HCl buffer, and the measurements of GDH activity were performed at the physiological pH 7.4, which is not optimal for this enzyme, but at which this enzyme acts in the blood. According to the Sigma-Aldrich data on the pH dependence of the reaction (1), the efficiency of the enzyme at a pH of 7.4 is lower than the maximum by about 5.5%^32^. The reaction mixture (330 μl), containing potassium phosphate buffer (pH 7.4), NADPH, EDTA, NH_4_Cl, and lysate (10 μl), was added into each well and incubated for 5 min at 30 °C, then the reaction was started by adding α-ketoglutarate. The final mixture in the well (340 μl) contained 50 mM potassium phosphate buffer (pH 7.4), 0.25 mM NADPH, 0.85 mM EDTA, 220 mM NH_4_Cl, 7.6 mM α-ketoglutarate and lysate (10 μl). GDH activity was measured by the decrease in optical density as a result of the NADPH conversion to NADP in the presence of α-ketoglutarate, NH_4_Cl, and GDH.

ALT activity was measured according to the procedure described by Bergmeyer et al.^33^, using two coupled reactions: (1) conversion of ALA and α-ketoglutarate by ALT to PYR and GLU, and (2) conversion of the formed PYR in the presence of NADH and lactate dehydrogenase (LDH) to LAC and NAD^+^. A reaction mixture (330 μl), containing Tris buffer (pH 7.4), alanine, pyridoxal 5′-phosphate, NADH, 10 μl lysate and lactate dehydrogenase (LDH) was incubated for 10 min at 30 °C. During this preliminary period of incubation NADH can be oxidized by ALT (in the presence of pyridoxal 5′-phosphate) due to the presence of pyruvate and other endogenous substrates in the sample. The reaction was then started by the addition of α-ketoglutarate The total volume of the reaction mixture in the measuring well was 340 μl. The final mixture in the well contained 100 mM Tris buffer (pH 7.4), 500 mM alanine, 0.1 mM pyridoxal 5′-phosphate, 0.18 mM NADH, 1.2 IU/ml lactate dehydrogenase, 15 mM AKG and 10 μl lysate (sample).

### Calculation of enzyme encapsulation and cell yield

The percentage of encapsulation and cell recovery were selected in this study as characteristics of the enzyme encapsulation efficiency. These indicators were calculated by the Eqs. (5, 6):

5 $${\text{Encapsulation }}\left( {{\text{E}}, \, \% } \right) \, = {\text{ A}}_{{{\text{amm}}}} \times {\text{V}}_{{{\text{amm}}}} \times {1}00/\left( {{\text{A}}_{{{\text{initial}}}} \times {\text{V}}_{{{\text{initial}}}} } \right),$$

6 $${\text{Cell}}\,{\text{recovery }}(\text{C},\% ) = {\text{V}}_{{{\text{amm}}}} \times {\text{Ht}}_{{{\text{amm}}}} \times 100/\left( {{\text{V}}_{{{\text{initial}}}} \times {\text{Ht}}_{{{\text{initial}}}} } \right),$$

where A_initial_ and A_amm_ are enzyme activities in suspensions of initial RBCs and final ammocytes, respectively; V_initial_ and V_amm_ are the volumes, and Ht_initial_ and Ht_amm_ are the hematocrits of these suspensions, respectively.

### Sample preparation for measuring the kinetics of ammonium consumption and alanine production

The rates of ammonium consumption and alanine production were measured in 3 ml samples placed in a hermetically sealed cuvette. The sample contained a buffer solution (pH 7.4) with 130 mM sodium phosphate, 10 mM glucose, 2.7 mM KCl, 1 mM MgCl_2_, 0.2 mM α-ketoglutarate, 10 mM sodium pyruvate, 1 mM NH_4_Cl supplemented by a suspension of ammocytes (up to hematocrit in the cuvette 23%). Native erythrocytes (without the encapsulated enzymes) were used as controls. The measurements were carried out at 25 °C for 120–140 min. Every 20 min, 100 μl of the sample was added to 100 μl of a mixture of 6% HClO_4_ and 40% ethanol. The extracts were thoroughly mixed and frozen at − 20 °C.

### Ammonium and alanine measurements

On the day of measurement, the extracts were quickly thawed and centrifuged for 5 min at 10,000*g* to remove insoluble sediment. Supernatant was taken, and 2 M К_3_PO_4_ was added to it to pH 6. Neutralised extracts were frozen again, thawed, and centrifuged for 5 min at 10,000*g* to remove the salt precipitate. Ammonium and alanine were measured fluorometrically using an AF2200 Plate Reader (Eppendorf AG, Germany) at excitation and NADH emission wavelengths of 365 and 465 nm, respectively.

Ammonium was measured according to the method of Nazar and Schoolwerth, using the conversion of α-ketoglutarate to glutamate in the presence of ammonium and NADH using GDH^34^. The final reaction mixture contained 0.2 M K-phosphate buffer pH 7.6, 60 μM NADH, 0.5 mM EDTA, 5 IU/ml GDH, 6 mM α-ketoglutarate. This blank solution was incubated for 30–60 min at 25 °C until the fluorescence level became constant. The reaction was started by adding 10 μl of extracts to each well for a final volume of 340 μl. After 1 h of incubation at 25 °C (with periodic shaking of the plate), the final fluorescence was measured. The ammonia concentration was calculated from the decrease in fluorescence using a standard fluorescence curve for various ammonia concentrations.

Alanine was measured by an increase in NADH fluorescence according to reaction catalyzed by alanine dehydrogenase (7) described in Ref.^35^:

7 $${\text{L - Alanine }} + {\text{ NAD}}^{ + } + {\text{ H}}_{{2}} {\text{O }} \to {\text{ pyruvate }} + {\text{ NADH }} + {\text{ NH}}_{{4}}^{ + } .$$

The final mixture contained buffer (40 mM Tris, 1 M hydrazine, 1.4 mM EDTA, pH 10.0, fresh prepared), 1 mM β-NAD, and 1.5 IU/ml ADH. This blank solution was incubated for 30–60 min at 25 °C until the fluorescence level became constant. The reaction was started by adding 30 μl of extracts to each well to the final volume of 340 μl. After 1 h of incubation at 25 °C (with periodic shaking of the plate), the final fluorescence was measured. Alanine concentration was calculated from the increase in fluorescence using a standard curve of alanine concentrations.

### Ammocytes and native RBCs storage

To study the properties of ammocytes during storage, samples of native cells (as a control) and samples of ammocytes were resuspended to a final hematocrit of 10% in a solution containing 137 mM NaCl, 2.7 mM KCl, 10 mM Na_2_HPO_4_, 2 mM KH_2_PO_4_, 1.3 mM CaCl_2_, 5 mM MgCl_2_, 10 mM glucose, 5% (w/w) BSA, 30 mM HEPES, 0.28 mM adenine, and 0.02 mg/ml ampicillin (pH 7.4). These suspensions were stored at 4 °C for 6 days. Erythrocyte indices, hemolysis, osmotic fragility, as well as intracellular and extracellular activity of GDH and ALT were studied. The deformability of the native erythrocytes and obtained ammocytes was measured immediately after preparation. For ammocytes, the deformability was also measured after 2 h of incubation in the same solution at room temperature.

### Osmotic fragility test

The measurements were carried out according to Shcherbachenko et al.^36^. In the wells of a 96-well plate, 300 μl of solutions with different osmolality (from 0 (distilled water) to 297 mOsm/kg, pH 7.4) were added. To each well was added 6 μl of the sample (erythrocyte suspension diluted in PBS to 5% hematocrit). The mixtures were incubated for 30 min at room temperature. Then the osmolality of the solutions was returned to normal by adding to each well 40 μl of a hypertonic solution containing 96.2 mM Na_2_HPO_4_, 15.6 mM NaH_2_PO_4_, 1.54 M NaCl (pH 7.4). Light scattering of the samples was measured at 620 nm using an Anthos Zenyth 340rt microplate photometric reader (Biochrom, England). The percent of non-lysed cells was determined as the ratio of the optical density measured in a particular sample to the optical density of the same sample without lysis (in a solution with physiological osmolality). The value of osmotic fragility was characterised by the osmolality of the solution in which 50% of the original cells were lysed (H_50_). Also, the width of the cells’ distribution in accordance with the lysis capacity (W) was calculated, which was determined as the difference in the osmolality of solutions in which 10 and 90% of cells were lysed.

### Hemolysis

To assess spontaneous hemolysis, samples were taken from RBCs suspensions with known hematocrit and centrifuged for 8 min at 1000*g*. Supernatants were diluted 10 times with water, and initial erythrocyte suspensions were diluted 100 times. The optical density of hemoglobin was measured in 1 ml cuvettes at a wavelength of 540 nm. Hemolysis was calculated by the Eq. (8):

8 $${\text{G}} = \left[ {\left( {{1} - {\text{Ht}}_{{{\text{RBC}}}} } \right) \times {\text{OD}}_{{{\text{supernatant}}}} \times {\text{df}}_{{{\text{supernatant}}}} } \right] \times {1}00/{\text{OD}}_{{{\text{RBC}}}} \times {\text{df}}_{{{\text{RBC}}}} ,$$

where G is hemolysis (%), Ht_RBC_ is suspension hematocrit (%), OD_RBC_ and OD_supernatant_ are optical densities in samples of erythrocyte suspension and supernatant, respectively, df_supernatant_ and df_RBC_ are dilution factors of supernatant and RBC suspension, respectively.

### Erythrocyte indices

Standard erythrocyte indices for native erythrocytes and erythrocyte-bioreactors were measured using a Micros OT automatic hematology analyzer (ABX-France).

### Osmolality of solutions

Osmolality of solutions was measured using OSKR-1M osmometer (KIWI, Russia).

### Cell filterability

Cell filterability was measured by a simple filtration method (based on a modified method used in the Hanss hemorheometer) described by Lisovskaya et al.^37^. Suspension samples were diluted with PBS to a hematocrit of 0.1%. The measurements were carried out at room temperature. The time required to pass through the filter a fixed volume (0.250 ml) of PBS (t_b_) and then the erythrocyte suspension (t_s_) was measured with an accuracy of 0.1 s using a sensor installed in the lid covering the sample-filled column. A 7 μm thick polyethylene terephthalate film with an average pore diameter of 3.1 μm was used as a membrane filter (Joint Institute for Nuclear Research, Dubna, Moscow Region, Russia). Two parameters were defined: the index of filterability F:

9 $${\text{F}} = {\text{t}}_{{\text{b}}} /{\text{t}}_{{\text{s}}} ,$$

and the proportion of cells that cannot pass through the filter (Z, %), which was calculated according to Ref.^37^, based on the known total number of filter pores (N_0_) and the number of RBCs that were placed on the filter (m) using the Eq. (10):

10 $${\text{Z}} = {\text{N}} \times {1}00/{\text{m,}}$$

where N is the number of pores that were blocked by unfiltered cells.

The value of N was determined using the re-passage time of the buffer (0.250 ml) through the filter (t_b1_) after 0.250 ml of the test suspension was passed through this filter and the filter was washed (11):

11 $${\text{N }} = {\text{ N}}_{0} \times \left( {{\text{t}}_{{{\text{b1}}}} - {\text{t}}_{{\text{b}}} } \right)/{\text{t}}_{{{\text{b1}}}} .$$

### Statistical data analysis

The distribution of all the experimental parameters was normal (according to the D'Agostino-Pearson test (MedCalc Statistical Software bvba, version 14.12, Ostend, Belgium)). All the experimental results were analyzed using one-way ANOVA and are presented as the mean ± standard deviation (SD), except certain cases, which are indicated. The difference was considered significant at p < 0.05. Statistical data analysis was performed using the OriginPro 8.1 software (OriginLab Corporation, Northampton, MA, USA).

## Data Availability

All data generated or analysed during this study are included in this published article.
